# From symptom discovery to treatment - women's pathways to breast cancer care: a cross-sectional study

**DOI:** 10.1186/s12885-018-4219-7

**Published:** 2018-03-21

**Authors:** Jennifer Moodley, Lydia Cairncross, Thurandrie Naiker, Deborah Constant

**Affiliations:** 1 Cancer Research Initiative, Faculty of Health Sciences, University of Cape Town, Anzio Road, Observatory, 7925, Cape, Town, South Africa; 2 Women’s Health Research Unit, School of Public Health and Family Medicine, Faculty of Health Sciences, University of Cape Town, Anzio Road, Observatory, 7925, Cape, Town, South Africa; 3 SAMRC Gynaecology Cancer Research Centre, Faculty of Health Sciences, University of Cape Town, Anzio Road, Observatory, 7925, Cape, Town, South Africa; 4 Department of Surgery, University of Cape Town, Anzio Road, Observatory, 7925, Cape, Town, South Africa; 5 Department of Radiation Oncology, University of Cape Town, Anzio Road, Observatory, 7925, Cape, Town, South Africa

**Keywords:** Breast cancer, Cancer symptoms, Timely diagnosis, Delay in diagnosis, Breast cancer knowledge, South Africa

## Abstract

**Background:**

Typically, women in South Africa (SA) are diagnosed with breast cancer when they self-present with symptoms to health facilities. The aim of this study was to determine the pathway that women follow to breast cancer care and factors associated with this journey.

**Methods:**

A cross-sectional study was conducted at a tertiary hospital in the Western Cape Province, SA, between May 2015 and May 2016. Newly diagnosed breast cancer patients were interviewed to determine their socio-demographic profile; knowledge of risk factors, signs and symptoms; appraisal of breast changes; clinical profile and; key time events in the journey to care. The Model of Pathways to Treatment Framework underpinned the analysis. The total time (TT) between a woman noticing the first breast change and the date of scheduled treatment was divided into 3 intervals: the patient interval (PI); the diagnostic interval (DI) and the pre-treatment interval (PTI). For the PI, DI and PTI a bivariate comparison of median time intervals by various characteristics was conducted using Wilcoxon rank-sum and Kruskal-Wallis tests. Cox Proportional-Hazards models were used to identify factors independently associated with the PI, DI and PTI.

**Results:**

The median age of the 201 participants was 54 years, and 22% presented with late stage disease. The median TT was 110 days, with median patient, diagnostic and pre-treatment intervals of 23, 28 and 37 days respectively. Factors associated with the PI were: older age (Hazard ratio (HR) 0.59, 95% CI 0.40–0.86), initial symptom denial (HR 0.43, 95% CI 0.19–0.97) and waiting for a lump to increase in size before seeking care (HR 0.51, 95% CI 0.33–0.77). Women with co-morbidities had a significantly longer DI (HR 0.67, 95% CI 0.47–0.96) as did women who mentioned denial of initial breast symptoms (HR 4.61, 95% CI 1.80–11.78). The PTI was associated with late stage disease at presentation (HR 1.78, 95% CI 1.15–2.76).

**Conclusion:**

The Model of Pathways to Treatment provides a useful framework to explore patient’s journeys to care and identified opportunities for targeted interventions.

**Electronic supplementary material:**

The online version of this article (10.1186/s12885-018-4219-7) contains supplementary material, which is available to authorized users.

## Background

Breast cancer, the commonest cancer among women worldwide, is a major and growing public health burden. Incidence rates have increased steadily since 2008 and currently 1.7 million new cases are diagnosed each year [1, 2]. In 2012 the majority (53%) of new breast cancer cases were among women living in low- and middle-income countries (LMICs) [1, 2], where the shift toward more affluent lifestyles, particularly those associated with dietary and reproductive risk factors, has been associated with a rising burden of cancers. Lack of early detection programs and poor access to treatment, place women in LMICs at a high cancer mortality risk.

Breast cancer is the commonest cancer among women in South Africa (SA) with an age-standardized incidence rate of 35 per 100,000 women [3]. SA does not have a national mammography-screening program. Typically, women with breast symptoms self-present to primary health care facilities and are referred to secondary or tertiary level health facilities if further diagnostic work-up and treatment is required [4]. Minimizing time to diagnosis is dependent on timely presentation to primary health care providers by women with symptoms suggestive of breast cancer; appropriate assessment at the primary health care level and; timely access to referral and treatment centres. For people with potential symptoms of cancer the journey to cancer diagnosis is complex and influenced by a multitude of factors including: knowledge and awareness of cancer symptoms; the nature of the symptoms; perception of risk and; health system, psychological, and socio-cultural barriers to health care [5–14]. Understanding the influence of these factors on the pathway women follow to breast cancer diagnosis is vital to the development of locally relevant, targeted interventions. Little is known about the pathway that women follow to breast cancer diagnosis and treatment in SA.

Theoretical frameworks provide a systematic approach to understanding health behavior and there have been calls for greater theoretical underpinning of help-seeking research [15–17]. The Model of Pathways to Treatment provides a useful research framework to explore and understand patient’s journeys as it takes into account the complex and dynamic nature of help-seeking behavior [16, 17]. The Model identifies five key events in the pathway to care: detection of bodily changes; perceived reasons to discuss symptoms with a health care provider; first consultation with a health care provider; diagnosis and start of treatment, and four important intervals between these events: the appraisal, help-seeking, diagnostic and the pre-treatment intervals [16]. The Model also identifies 3 main types of contributing factors that influence the timing of events and duration of intervals. These include patient (e.g. socio-demographics), health system (e.g. access to health care) and disease-related factors (e.g. site, growth-rate). By increasing understanding of the factors influencing the key events in the pathway to care, the model can be used to identify targets for interventions in order to encourage early detection, presentation, and treatment. Using the Model of Pathways to Treatment framework, we explored patient’s pathways from breast cancer symptom discovery to treatment, detailing time intervals and factors influencing these intervals.

## Methods

### Study design, study site and sample size

A cross-sectional study was conducted at a tertiary hospital in the Western Cape Province, SA, between May 2015 and May 2016. The hospital has an open-access, one-stop diagnostic breast clinic where women may present with a letter from a primary level provider (nurse practitioner or doctor). The breast clinic provides a same day clinical and cytological evaluation with referral to the Combined Breast Clinic (CBC) if the breast cytology is positive for malignancy. Participants were recruited from the CBC where a multidisciplinary clinical team of surgeons, oncologists, radiologists and pathologists, review new patients to make a definitive breast cancer diagnosis and develop a management plan.

Based on clinic records we anticipated that 500 new breast cancer patients would be seen over a 1-year period. The sample size calculation was based on the proportion of women expected to have a > 3-month duration from symptom discovery to treatment (delayed presentation). Based on the literature we predicted that 60% of women would have a delayed presentation. To estimate the proportion to within 5% of the true value with a 95% confidence interval, a sample size of 213 was required.

### Data collection

Breast cancer patients were interviewed within 2 weeks of diagnosis by a trained interviewer using a structured questionnaire (Additional file 1), with relevant clinical information retrieved from the hospital records. Information was collected on: the socio-demographic profile of participants; knowledge of risk factors, signs and symptoms with questions derived from the Breast Cancer Awareness Measure [18]; breast habits and beliefs; appraisal of breast changes; the clinical profile and; key time events in the journey to diagnosis and care.

Socio-demographic details included: age; main home language; educational level; employment status; medical aid membership; marital status and; household income status as classified by the hospital (H0 = persons on social grant; H1 = individual income < $5366 per annum (p.a.) or family income < $7667 p.a.; H2 = individual income between $5367 and $19,168 p.a. or family income between $7667 and $26,852 p.a.; and H3 = individual income >$19,168 p.a. or family income > $26,852 p.a.)

Knowledge of breast cancer risk factors and signs was ascertained by first asking an unprompted open-ended question “Can you name as many risk factors of breast cancer/signs of breast cancer that you can think of?” This was followed by a set of prompted closed questions to determine knowledge of specific risk factors and signs e.g. Can you tell me if you think a lump in the breast is a sign of breast cancer? Participants were asked to respond with a “Yes, No or Don’t Know” to each specific closed question. An unprompted composite knowledge score was computed by combing the score for unprompted knowledge of risk factors and the score for unprompted knowledge of signs of breast cancer, with a maximum score of 22. The categories for unprompted knowledge were based on the distribution of the scores and created so as to represent the upper and lower ranges of scores as well as the central tendency. The highest score for unprompted composite knowledge was 13. Based on the distribution of the scores, unprompted composite knowledge was divided into 3 balanced categories: no knowledge (score 0), very little knowledge (score < 4) and little knowledge (score ≥5).

Breast habits, symptom appraisal and pathways to care variables and response options included: history of breast self-examination (Yes/No); type of first breast change noticed (open question responses coded as lumps in the breast, lumps in the armpit, bleeding or discharge from nipple, nipple changes, changes in breast skin, changes in size or shape of breast, pain in breast or armpit, other); appraisal of breast change (open question responses coded as mention (Yes/No) of: breast cancer, not serious, ignored symptom, denial, other); reason to have symptom checked (open question responses coded as mention (Yes/No) of: lump increasing in size, pain, prompted by family/friend, prompted by reading pamphlet/breast awareness event/television program, concerned about changes/wanted to make sure nothing was wrong, other); type of first health care provider seen(public sector provider, private sector provider, other).

Clinical variables included: family history of breast cancer; menopausal status; parity; breastfeeding; hormonal contraceptive and hormonal replacement use; alcohol and smoking habits; and participant reported history of benign breast disease or of any co-morbidity e.g. hypertension, diabetes mellitus. In addition, the following information was abstracted from the clinical records: histological diagnosis, hormonal receptor status (estrogen (ER), progesterone (PR) and human epidermal growth factor (HER2)); and the tumour, node and metastasis (TNM) status at diagnosis which was used to classify patients as having early (I, II) or late stage (III or IV) disease.

The total time (TT) between a woman noticing the first breast change and the date of scheduled treatment for breast cancer was divided into 3 intervals: the patient interval (PI) defined as the time between date of first breast change to date of first health care provider consultation; the diagnostic interval (DI) which was the time between the first health care provider visit and the date of diagnosis and; the pre-treatment interval (PTI), defined as the time between the date of diagnosis and the date treatment was due to commence. A calendar prompt was used to assist participants’ memory of key dates and events. Date of diagnosis was defined as the date that a clinical diagnosis was made at the CBC.

### Data analysis

Data was entered into a Microsoft Access database and analyzed using STATA v.13. Descriptive statistics (means, medians, proportions) were used to characterize the variables. Crude bivariate comparisons (using Wilcoxon rank-sum and Kruskal-Wallis tests to compare medians and ranked distributions, and Yates corrected chi-square and Fisher’s Exact test to compare proportions) were used to identify factors associated with stage at presentation (early versus late). Multivariate logistic regression was used to identify independent factors associated with late stage presentation. Variables included in the model were those significant with bivariate analysis and those of a priori interest. The final model included: age (categorized using the median); educational level; marital status; employment status; composite unprompted knowledge score, first symptom (lump vs. other) and mention of pain as a reason for seeking care.

Crude bivariate comparisons (using Wilcoxon rank-sum and Kruskal-Wallis tests to compare medians and ranked distributions, and Yates corrected chi-square and Fisher’s Exact test to compare proportions) were used to identify factors associated with a total time from first change to scheduled treatment of > 3 months. Multivariable logistic regression analysis was conducted to determine independent predictors of a total time of > 3 months. Variables significant with analysis (*p* < 0.05), and those of a priori interest were included in the model: age (categorized using the median); educational level; composite unprompted knowledge score; first change noticed (breast lump or other); type of health care provider seen (public sector, private sector or other); mention of increase in lump size as a reason for seeking care; mention of concern about breast changes as a reason for seeking care and; stage at presentation.

For the PI, DI and PTI a bivariate comparison of median time intervals by various characteristics was conducted using Wilcoxon rank-sum and Kruskal-Wallis tests to compare medians and ranked distributions. Cox Proportional-Hazards models were used to establish factors independently associated with the PI, DI and PTI. All Cox regression models included variables that were significant with bivariate analysis (*p* < 0.05), and variables of a priori interest. For the PI regression model variables included were: age (categorized using the median); educational level; marital status; unprompted composite knowledge score; history of co-morbidities (benign breast disease, any other co-morbidity, no co-morbidity); stage of disease (early or late); first change noticed (breast lump or other); thought first change was breast cancer; ignored first breast change; thought first change was minor/not serious; mention of family or friends as a being a reason for seeking care; mention of increase in lump size as a reason for seeking care and; mention of concern about breast change as a reason for seeking care. For the DI regression model variables included were: age (categorized using the median); educational level; history of co-morbidities (benign breast disease, any other co-morbidity, no co-morbidity); first change noticed (breast lump or other); mention of denial as initial response to breast change; stage of disease (early or late) and; type of health care provider first seen (public or private sector). The PTI regression model included the following variables: age (categorized using the median); educational level; stage of disease (early or late) and type of first treatment (surgery or other).

Ethical approval to conduct the study was obtained from the Human Research Ethics Committee, University of Cape Town (Reference number 313/2013). Written informed consent was obtained from all participants.

## Results

A total of 216 women were approached to participate in the study: 8 refused (1 due to time constraints, 1 did not feel emotionally ready, 5 were not interested in the research study) and 7 were ineligible. The median age of the 201 participants was 54 years, interquartile range (IQR) 45–63. Table 1 outlines the socio-demographic profile of the participants. The majority of women had a high school or higher educational level and 75% were unemployed.

**Table 1:** Socio-demographic profile of participants

Characteristic	Total (201) n (%)
Main Home Language	
English	68 (33.8)
Afrikaans	80 (39.8)
Xhosa	45 (22.4)
Other	8 (4.0)
Education level	
None-Grade 7	49 (24.4)
Grade 8-Grade 11	96 (47.8)
Grade 12+	56 (27.9)
Marital status	
Married	84 (41.8)
Single in stable relationship	6 (3.0)
Single	42 (20.9)
Widowed	38 (18.9)
Divorced/separated	31 (15.4)
Employed	51 (25.4)
Have medical insurance	6 (3.0)
Ever smoked	45 (22.5)
Ever drank alcohol	12 (6.2)
Household income status^a^	
H0	66 (32.8)
H1	81 (40.3)
H2	32 (15.9)
H3	19 (9.5)

^a^3 records missing.

*H0*: persons on social grant; *H1*: individual income < $5366 per annum (p.a.) or family income < $7667 p.a.; *H2*: individual income between $5367 and $19,168 p.a. or family income between $7667 and $26,852 p.a.; and *H3*: individual income >$19,168 p.a. or family income > $26,852 p.a

### Knowledge of breast cancer risk factors and signs

**Fig. 1:**
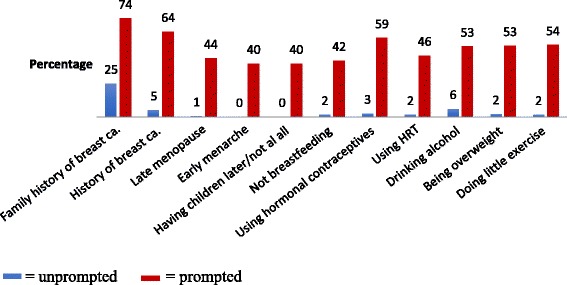
Unprompted and prompted knowledge of breast cancer risk factors

**Fig. 2:**
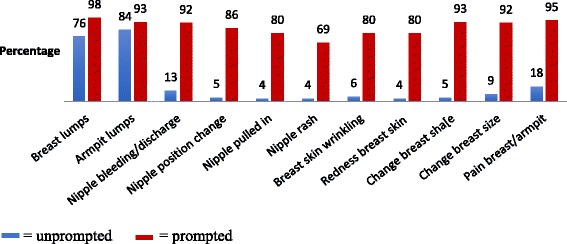
Unprompted and prompted knowledge of breast cancer signs

Women had very limited knowledge of breast cancer risk factors. In response to the unprompted question i.e. “Can you name as many risk factors of breast cancer that you can think of,” 67% of woman could not name a single risk factor. The most commonly recognized risk factor was a family history of breast cancer: 25% and 74% of women in the open and closed question respectively (see Fig. 1). Most women were aware that a breast or armpit lump was a sign of breast cancer. When unprompted i.e. in response to the open question “Can you name as many signs factors of breast cancer that you can think of”, knowledge of other breast cancer signs was limited (see Fig. 2).

### Symptom appraisal

For the majority (74%) of women the first symptom noticed was a breast lump, 8% reported pain in the breast or armpit as the first symptom, 7% noticed a change in breast size, 4% reported nipple changes and 3% a lump in the armpit as the first change. Fifty percent of women appraised the first symptom as being minor or not serious, 31% thought it could be breast cancer and 4% reported being in denial. Once a symptom was noticed the main reasons for seeking health care included: wanting to make sure nothing was wrong (61%); persuasion by family members and friends (50%); pain (29%) and; because the lump was getting bigger (25%). The majority (72%) of women first had their symptom assessed at a public sector primary health care service.

### Clinical history and profile

A history of co-morbidities was fairly common: 47% of women gave a history of hypertension, 13% had benign breast disease and 12% reported having diabetes. Thirty-eight percent of women had a family history of breast cancer. Just over half (55%) of the women interviewed stated that they were in the habit of checking their breasts, with the vast majority of these women reporting breast self-examination at least once a month. The commonest histological subtype observed was invasive ductal carcinoma (77%) and 14% of women had triple negative disease. Twenty-two percent of women presented with late stage (stages III and IV) disease. Using bivariate analysis being single vs. being married [15 (36%) vs. 18 (21%), *p* = 0.034], first symptom not being a lump vs. lump [19 (37%) vs. 25 (17%), *p* = 0.004] and mention of pain as a reason for seeking care vs. not mentioned [19 (33%) vs. 25 (18%), p = (0.019] were all associated with late stage at presentation. On multivariate analysis, none of these factors remained significant at the 0.05 cut-off level (being single vs. being married adjusted Odds Ratio (aOR) 2.20, 95% CI 0.89–5.42, *p* = 0.087; first symptom not being a lump vs. lump aOR 0.47, 95% CI 0.21–1.04, *p* = 0.064; mention of pain as a reason for seeking care vs. not mentioned aOR 1.97, 95% CI 0.88–4.41, *p* = 0.097).

### Key time intervals in the pathway to diagnosis and treatment

The overall median time from first symptom discovery to initiation of treatment was 110 days (IQR 67–178). For 60% of patients the time between symptom discovery and treatment initiation exceeded 3 months. Compared to those with shorter time from symptom discovery to treatment i.e. ≤ 3 months), those with a longer interval were significantly more likely to mention seeking care because the lump was getting bigger aOR 2.7 95% confidence interval (CI) 1.15–6.12) and less likely to mention that they sought medical care because they were worried about the initial changes (aOR 0.30, 95% CI 0.15–0.61). Details on the patient, diagnostic and pre-treatment intervals and associated factors are provided below.

### Patient interval

The median patient interval was 23 days, IQR 6–64 days. Women whose interpretation of the initial symptom as possibly being breast cancer and women who mentioned concern about the symptom as a reason for seeking care had a significantly shorter median PI (Table 2). Initial denial of symptoms, appraising the symptom as minor, being prompted by family members or friend to seek care and only seeking care when a lump increased in size were associated with significantly longer PI intervals (Table 2). On regression analysis older age (HR 0.59, 95% CI 0.40–0.86); those who initially denied symptoms (HR 0.43, 95% CI 0.19–0.97); those who sought care to check that nothing was wrong (HR 1.76, 95% CI 1.20–2.58) and those waited for a lump to increase in size before seeking care (HR 0.51, 95% CI 0.33–0.77) were significant factors (see Additional file 2).

**Table 2:** Association between participant characteristics and patient interval (*n* = 187)

Variable	Patient interval		
	N	Median	*p*-value
Age^a^			
Age ≤ 54 years	95	21	0.355
Age > 54 years	90	29	
Education			
None-Grade 7	43	35	0.807
Grade 8-Grade 11	91	22	
Grade 12+	53	23	
Marital status			
Married	76	31	0.163
In stable relationship	6	10	
Single	41	15	
Widowed	35	26	
Divorced/separated	29	33	
Paid work			
Yes	48	15	0.064
No	139	28	
Income status^b^			
H0	59	32	0.668
H1	76	17	
H2	30	19	
H3	19	33	
Composite unprompted knowledge			
None	14	21.5	0.308
Very little	160	23	
Little	13	19	
History of co morbidities			
Benign breast disease/fibroadenoma	23	23	0.522
Any other co-morbidity	104	22	
None	60	32	
Family history of breast cancer			
Yes	74	23	0.953
No	97	23	
Not sure	16	19	
Habit of checking breasts for lumps/changes			
Yes	105	20	0.087
No	82	31	
First breast change notice			
Lump	140	22	0.785
Other changes	47	23	
Appraisal of first change			
Thought it was not serious/minor			
Mentioned	92	33	0.027
Not mentioned	95	15	
Thought it was breast cancer			
Mentioned	59	12	0.005
Not mentioned	128	31	
Not sure what change meant			
Mentioned	109	27	0.413
Not mentioned	78	20	
Ignored it/was in denial			
Mentioned	7	111	0.003
Not mentioned	180	22	
Reason to have symptom checked			
Felt pain^c^			
Mentioned	54	27	0.550
Not mentioned	132	22	
Lump was increasing in size^c^			
Mentioned	47	52	0.001
Not mentioned	139	15	
Prompted by family/friends^c^			
Mentioned	92	31.5	0.053
Not mentioned	94	15	
Prompted by pamphlet/breast cancer awareness event/TV program^c^			
Mentioned	50	25	0.719
Not mentioned	136	22	
Wanted to be sure nothing was wrong^c^			
Mentioned	112	18	0.046
Not mentioned	74	34	
First health care provider seen			
Public sector primary health care clinic or district hospital	138	27	0.095
Private practitioner	45	12	
Other	4	45	
Cancer stage			
Early (stage 1&2)	144	20	0.199
Late (stage 3&4)	43	33	

^a^ 2 records missing, ^b^ 3 records missing, ^c^ 1 record missing.

*H0*: persons on social grant; *H1*: individual income < $5366 per annum (p.a.) or family income < $7667 p.a.; *H2*: individual income between $5367 and $19,168 p.a. or family income between $7667 and $26,852 p.a.; and *H3*: individual income >$19,168 p.a. or family income > $26,852 p.a

### Diagnostic interval

The median time between the first health care visit and a breast cancer diagnosis was 28 days (IQR 13–58 days). Fifty-four percent of women had made 4 or more health care visits between symptom discovery and a breast cancer diagnosis, whilst 11% made 6 or more visits. Using bivariate analysis (Table 3) women who first appraised the symptom as being minor compared to those that did not (32 days vs. 22 days, *p* = 0.047) and, women with a past history of benign breast disease, or a history of other co-morbidities had a significantly longer diagnostic interval compared to those with no co-morbidities (median interval 48, 29 and 20 days respectively, *p* = 0.004); whilst women whose initial reaction was denial of the breast symptom had a significantly shorter diagnostic interval (11 days vs. 29 days, *p* = 0.010). When adjusted for other factors (Cox regression analysis), a history of other co-morbidities HR 0.67, 95% CI (0.47–0.96) and denial of initial breast symptoms (HR 4.61, 95% CI 1.80–11.78) remained significant (see Additional file 3).

**Table 3:** Association between participant characteristics and diagnostic interval (*n* = 193)

Variable	Diagnostic interval		
	N	Median	*p*-value
Age^a^			
Age ≤ 54 years	95	23	0.370
Age > 54 years	96	30	
Education			
None-Grade 7	48	30	0.839
Grade 8-Grade 11	93	23	
Grade 12+	52	25	
Marital status			
Married	82	29	0.673
In stable relationship	6	15	
Single	40	23	
Widowed	34	28	
Divorced/separated	31	22	
Paid work			
Yes	50	27	0.654
No	143	28	
Income status^b^			
H0	63	34	0.757
H1	76	23	
H2	32	27	
H3	19	22	
Composite unprompted knowledge^a^			
None	16	24	0.300
Very little	164	25	
Little	13	34	
History of co-morbidities			
History of benign breast disease/fibroadenoma	24	48	0.004
Any other co-morbidity	105	29	
None	64	20	
Family history of breast cancer			
Yes	75	23	0.635
No	100	28	
Not sure	18	35	
Habit of checking breasts for lumps/changes			
Yes	108	30	0.172
No	85	25	
First breast change noticed^c^			
Lump in breast	140	23	0.155
Other changes	49	34	
Appraisal of first change			
Thought it was not serious/minor^c^			0.047
Mentioned	94	31.5	
Not mentioned	95	22	
Thought it was breast cancer ^c^			
Mentioned	58	22	0.346
Not mentioned	131	29	
Not sure what change meant^c^			
Mentioned	115	27	0.206
Not mentioned	74	26	
Ignored it/was in denial			
Mentioned	6	11	0.010
Not mentioned	187	29	
Reason to have symptom checked			
Felt pain^d^			
Mentioned	56	30	0.443
Not mentioned	132	23	
Lump was increasing in size^d^			
Mentioned	47	23	0.459
Not mentioned	141	29	
Prompted by family/friends^d^			
Mentioned	94	23	0.486
Not mentioned	94	30	
Prompted by pamphlet/breast cancer awareness event/TV program^d^			
Mentioned	48	21	0.317
Not mentioned	140	29	
Wanted to be sure nothing was wrong^d^			
Mentioned	116	23	0.180
Not mentioned	72	34	
First health care provider seen^b^			
Public sector primary health care clinic or district hospital^b^	138	30	0.510
Private practitioner	48	24	
Other	4	14	
Cancer stage			
Early (stage 1&2)	154	28	0.825
Late (stage 3&4)	39	21	

^a^ 2 missing records, ^b^ 3 missing records, ^c^ 4 missing records, ^d^ 5 missing records.

*H0*: persons on social grant; *H1*: individual income < $5366 per annum (p.a.) or family income < $7667 p.a.; *H2*: individual income between $5367 and $19,168 p.a. or family income between $7667 and $26,852 p.a.; and *H3*: individual income >$19,168 p.a. or family income > $26,852 p.a

### Pre-treatment interval

The median time from diagnosis to date of scheduled treatment was 37 days, IQR 18–50 days. Women with late stage disease had a significantly shorter PTI compared to women with early stage disease (median 21 days vs. 40 days, *p* = 0.001), whilst women with surgery as opposed to other types of treatment had a longer PTI (median 40 days vs. 15 days, *p* = 0.002) and women whose first line of treatment was chemotherapy as compared to first modes of treatment had a shorter median PTI (14 days vs. 40 days, *p* < 0.001) (Table 4). When adjusted for other factors using Cox regression, late stage of disease at presentation remained significant (HR 1.78, 95% CI 1.15–2.76, *p* = 0.010), see Additional file 4.

**Table 4:** Association between participant characteristics and pre-treatment interval (*n* = 192)

Variable	Pre-treatment interval		
	N	Median	p-value
Age^a^			
Age ≤ 54 years	95	37	0.795
Age > 54 years	95	35	
Education			
None-Grade 7	47	21	0.044
Grade 8-Grade 11	92	41	
Grade 12+	53	40	
Marital status			
Married	83	42	0.344
In stable relationship	6	18	
Single	39	28	
Widowed	34	29	
Divorced/separated	30	37	
Paid work			
Yes	51	37	0.860
No	141	35	
Income status^b^			
H0	62	39	0.415
H1	76	33	
H2	32	35	
H3	19	30	
Co-morbidities			
History of benign breast disease/fibroadenoma	24	36	0.538
Any other co-morbidity	106	34	
None	62	40	
Cancer stage			
Early (stage 1&2)	154	40	0.001
Late (stage 3&4)	38	21	
First treatment surgery^c^			
Yes	157	40	0.002
No	34	15	
First treatment chemotherapy^c^			
Yes	27	14	< 0.001
No	164	40	
First treatment hormonal therapy^c^			
Yes	13	28	0.753
No	178	36	
First treatment radiotherapy^c^			
Yes	2	91	0.105
No	189	35	

^a^ 2 records missing, ^b^ 3 records missing, ^c^ 1 record missing.

## Discussion

Our study is the first in South Africa quantifying time intervals and associated factors between symptom detection and breast cancer treatment, using the Model of Pathways to Treatment as a guiding framework [16]. Key factors influencing the journey to care were: limited knowledge of breast cancer risk factors and signs; sub-optimal symptom interpretation and appraisal; waiting for symptoms to worsen before seeking care; and the presence of co-morbidities.

Across all cancer sites, non-recognition of the seriousness of cancer symptoms has been shown to be an important risk factor for delays in seeking care [19]. For breast cancer knowledge, awareness and risk perception all influence initial symptom interpretation [20]. Among our patients, knowledge of breast cancer risk factors and symptoms was low, pointing to a clear need for targeted interventions to improve knowledge as this may hasten help-seeking behavior. Despite this low level of knowledge of breast cancer symptoms, a significant proportion of women reported practicing regular breast self-examination. This finding is of concern as it would be assumed that programs promoting breast self- examination would also be teaching women about risk factors and all signs of breast cancer. Perhaps this finding should prompt a refocus of breast awareness campaigns to emphasize all signs of breast cancer rather than only emphasizing breast self-examination. This shift would be in keeping with the change in policy in the US and UK from promoting regular breast self-examination to promoting breast awareness [21, 22]. The incongruence between our findings of poor knowledge of signs and regular breast self-examination could also be due to social desirability bias. A recent Cochrane review reported that brief interventions have the potential to increase breast awareness among women, although further studies are required to validate this [23]. Encouragingly, a study in Malaysia demonstrated that a public breast cancer awareness program coupled with staff training reduced late stage presentation of breast cancer by half over a four- year period [24].

Time intervals are often reported on as “time delays” in the literature. This incorrectly can imply a decision for inaction; however, the extensive use of “time delay” in the literature makes it difficult to avoid the term in this report. The association between time from symptom detection to cancer treatment, often referred to as the total time delay, and survival is complex [25–28]. A landmark systematic review of 87 breast cancer studies showed that a delay of > 3 months was associated with worse survival, compared to treatment within 3 months of symptom detection (OR 1.47, 95% CI 1.42–1.53) [29]. Recent studies have however produced mixed results but many did not take into account differences in tumour growth and the confounding effect of lead-time bias [25, 30]. A further complication in interpreting the association between time intervals and outcome is the range of methods used to measure time points and events, making comparison of studies difficult. Whilst it may be difficult to quantify the benefit on survival, recognized benefits of earlier time to treatment include, earlier stage at diagnosis, decreased morbidity and symptom relief [26, 27], thus reducing time delays is of importance.

Studies on intervals to treatment for women with breast cancer show marked differences between LMIC and high-income countries (HIC)s. A review of time intervals for breast cancer patients in10 HICs and LMICs [28] showed that among HICs, the median total time interval ranged between 1 to 1.6 months with more than 60% of patients commencing treatment less than 3 months from discovery. In comparison, the median total time interval for LMICs was between 5.5 to 8 months with fewer than 30% of patients starting treatment within 3 months of symptom discovery [28] .In this report total time interval data were not available for the few African countries included in the review [28]. Another meta-analysis of delays in breast cancer diagnosis and treatment conducted in 12 LMICs reported a mean total delay time of 3.6 months [7] . Median time delays were not reported and no African country data was included in the latter analyses. The only African study describing total median breast cancer delay times was conducted in Rwanda and documented a total time delay of 15 months – the longest ever recorded in the literature [31]. In our study, the median total time from symptom discovery to scheduled treatment was 3.9 months (110 days/ 15.7 weeks), with 60% of patients commencing treatment after 3 months i.e. shorter than median total delay times reported for LMICs by Unger-Saldana et al. [28] and Pace et al. [31] but considerably longer than that reported for HICs. Factors associated with the delay of > 3 months were related to symptom appraisal and included waiting for symptom progression before seeking care and a lack of initial concern about the symptom. These results are consistent with findings in an earlier qualitative study conducted in our hospital setting in which women reported a period of monitoring symptoms before deciding to seek care and did not perceive their initial breast symptoms as abnormal [13].

For breast cancer, the PI i.e. the time between symptom discovery and first presentation to a health care provider, differs widely between HICs and LMICs, with intervals as low as 7 days (median) reported in the UK [32] and as high as 11.9 months (mean) in Sudan [12]. In comparison, the median PI in our study was 23 days. Factors significantly associated with a longer PI included older women, those reporting initial denial of symptoms and women who waited for their lump to increase in size before seeking care. All of these have been reported previously in the literature as being associated with a longer PI [20, 33]. Our findings suggest that interventions to decrease the PI need to target older women and stress the importance of immediate help-seeking for symptoms rather than adopting a wait-and-see approach.

The median time of 28 days between first presentation to a health facility and diagnosis (diagnostic interval) in our study is well within the SA policy recommendation of 60 days [4]. It is also much shorter than that reported in other LMICs: median of 94 days in Brazil [14]; median of 150 days in Rwanda [31]; mean of 70 days in KwaZulu-Natal (KZN) South Africa [34] and; mean of 78 days in 12 LMICs [7]. The difference in DI between our study and the only other SA study reporting on diagnostic delay [34] is likely, in part, to be related to the benefit of our breast clinic being an open-access clinic, not requiring staging and work-up pre-referral, which is required is the KZN setting. Our findings point to the benefit of specialist breast units for diagnosis and management of breast disease, together with easy access to multi-disciplinary provincial oncology units – an approach that is in keeping with the new SA breast cancer prevention and control policy [4]. Half of the participants in our study however still made 4 or more visits to a health care provider before being referred to the one-stop breast clinic at the tertiary hospital. Reasons for the multiple visits before referral need to be explored. Interestingly patients whose initial reaction was one of denial had a significantly shorter diagnostic interval i.e. time between symptom detection and diagnosis. Further research is required to confirm this finding and explore how patient’s reactions to symptoms influence provider referral patterns. Of concern is the fact that women with co-morbidities, who would have had greater contact with the primary health care services, had a longer diagnostic interval compared to those without co-morbidities. Research exploring primary health care providers’ knowledge of risk factors and symptoms as well as challenges in managing and referring patients with potential symptoms of breast symptoms could help identify potential targets for interventions to further reduce the diagnostic interval.

For our patients, the longest interval in their journey to treatment was the pre-treatment interval – defined as the time between diagnosis and scheduled treatment (median 37 days). In our study women who required surgery as their first mode of treatment, had significantly longer waiting times than those requiring other modes of treatment. Our median waiting time of 40 days for surgical treatment post diagnosis is within the waiting time of less than 6 weeks from first diagnostic visit to definitive treatment suggested by both the European Society of Breast Cancer Specialist [35] and the UK National Health Service Guidelines [36], but longer than the SA policy recommendation of 31 days [4]. The median waiting time to surgery of 40 days reported in our study is achieved by the use of extra breast cancer theatre lists (Breast Cancer Project Lists) which are run by the Breast Surgical Unit in partnership with Groote Schuur Hospital and Project Flamingo (a non-profit organization dedicated to improving access to breast cancer treatment). These Project Lists take place on weekends and public holidays and between 8 and 12 extra full day lists are done per year. In the absence of these voluntary extra lists, the waiting time for surgery using the state health sector theatre resources alone would be approximately 12 weeks (84 days) (personal communication L Cairncross, Groote Schuur Hospital). This waiting time also does not take into consideration the fact that most women who are eligible for breast reconstruction cannot be offered this service due to pressures on theatre resources. The recommendation of 31 days outlined in the SA breast cancer prevention and control policy would need a considerable injection of resources in order to be met by most public-sector institutions in SA.

Our study has limitations. Due to logistic constraints we were unable to recruit to target (target = 213, number recruited = 201), and results need to be interpreted with this in mind. Retrospective recall could have affected accurate reporting of symptoms and health seeking behavior in our study. We sought to minimize this through the use of a calendar prompt and conducting interviews soon after diagnosis. However, the timing of interviews could also have resulted in difficulty in putting the journey into perspective if women were not emotionally prepared for this. Further, interviews conducted in a hospital setting could have resulted in a social desirability bias with under-reporting of time delays and over reporting of desirable behavior such as breast self-examination. We recognize that the time intervals reported are unlikely to be representative of intervals seen in public sector settings in SA without a tertiary centre-based one-stop breast clinic. However, results point to the potential intervals that can be achieved with one-stop specialized breast units.

## Conclusion

The Model of Pathways to Treatment provides a useful framework to explore patient’s journeys to care. Our study identified targets for interventions that could improve time to diagnosis. These include interventions that: address women’s limited knowledge of breast cancer risk factors and symptoms; promote breast awareness; target older women; address denial; encourage prompt help-seeking behaviour and educate women not to wait for a lump to get bigger or be painful before seeking care.

## Additional files

Additional file 1: Pathways to breast cancer care questionnaire. (PDF 344 kb)

Additional file 2: Predictors of the Patient Interval. Table with results of the Cox Regression analysis (DOCX 17 kb)

Additional file 3: Predictors of the Diagnostic Interval. Table with results of the Cox Regression analysis (DOCX 15 kb)

Additional file 4: Predictors of the Pre-Treatment Interval. Table with results of the Cox Regression analysis (DOCX 14 kb)

## Abbreviations

aOR: Adjusted Odds Ratio
CBC: Combined Breast Clinic
CI: Confidence Interval
DI: Diagnostic Interval
HICs: High-income countries
HR: Hazard Ratio
IQR: Inter-quartile Range
LMICs: Low- and middle-income countries
p.a.: Per Annum
PI: Patient Interval
PTI: Pre-Treatment Interval
SA: South Africa
TT: Total Time
