# Supplementary material for: From symptom discovery to treatment - women's pathways to breast cancer care: a cross-sectional study
Source: BMC Cancer. 2018 Mar 21;18:312. doi: 10.1186/s12885-018-4219-7 (PMC5863383; doi:10.1186/s12885-018-4219-7)
Supplement: Supplementary file 1 — Pathways to breast cancer care questionnaire. (PDF 344 kb) [file 12885_2018_4219_MOESM1_ESM.pdf]

Pathways to breast cancer care questionnaire
English/Afrikaans

SECTION 1: Socio-Demographic Characteristics

READ: "Thank you for agreeing to talk with me. To start, I am going to ask you some questions about yourself. "/ "Dankie dat u ingestem het om met my te gesels. Om te begin gaan ek u vrae vra oor uself."

No.	Questions and filters	Coding categories	Code	Skip
101.	What is your date of birth?<br>*Wat is u geboorte datum?*	___ ___ / ___ ___ / ___ ___<br>DD MM YY\JJ		
102.	What is your main home language?<br>*Wat is u huistaal?*<br><br>**CIRCLE ONLY ONE RESPONSE**	English/Engels	1	
		Afrikaans/Afrikaans	2	
		Xhosa/Xhosa	3	
		Other (specify) Ander/spesifiseer:<br>______________________________	4	
103.	What is the highest level of schooling/education you have **completed**?<br>*Wat is die hoogste vlak van opleiding wat u voltooi het?*<br><br>**AFTER PARTICIPANT RESPONDS, CIRCLE ONE**	None – Grade 7 (standard 5)/Geen -Graad 7(standerd 5)	1	
		Grade 8-Grade 11 (standards 6-9)/Graad 8-11(standerd 6-9)	2	
		Grade 12 (matric)/ Graad 12 (matriek)	3	
		Univ/Technikon/College degree, diploma	4	
104.	Where were you born?<br>*Waar is u gebore?*	Town/City/ Dorp /: Stad___________________<br>Province/: Provinsie___________________________		
105.	Where do you live right now?<br>*Waar woon u nou?*<br><br>**PLEASE GET SUBURB/NEIGHBOURHOOD (E.G. ATHLONE, HANOVER PARK), NAME OF NEAREST TOWN/CITY (E.G. CAPE TOWN, WORCESTER ETC.)**	Suburb/Area/Section/ Voorstad/Gebied\Seksie:<br>__________________<br>Town/City/ Dorp /Stad: ___________________<br>Province/: Provinsie___________________________		
106.	What is your marital status?<br>*Wat is u huweliks status ?*<br><br>**NOTE TO INTERVIEWER: READ ALL RESPONSES AND CIRCLE ONLY ONE**	Married/ Getroud	1	
		Single in stable relationship/Enkel in n stabiele verhouding	2	
		Single/ Enkel	3	
		Widowed/Wedewee	4	
		Divorced/ Geskei	5	
		Other (please specify/)Ander (spesifieseer asseblief):<br>___________________________________	6	
107.	Do you have a job for which you are paid?<br>*Het u n werk waarvoor u betaal word?*	Yes/ Ja	1	
		No/Nee	0	
108.	Are you covered by medical aid?<br>*Is u gedek met n mediese fonds?*	Yes/Ja<br>**Specify name of medical aid**:___________________	1	
		No/Nee	0	

No.	Questions and filters	Coding categories	Code	Skip
108.	Have you heard of Pink Drive?<br>*Het u gehoor van Pink Drive?*	Yes/ Ja	1	
		No/Nee	0	
		Not sure/ nie seker nie	88	

SECTION 2: Knowledge and Perception of Risk Factors and Symptoms of Breast Cancer

READ: Now I would like to ask your views on breast cancer/ Nou will ek u graag vra oor u opinie oor borskanker.

No.	Questions and filters	Coding categories	Code YES	Code NO	Skip
201.	What do you think increases someone's chances of breast cancer? Please name as many risks/things as you can think of.<br>*Wat dink u vermeerder 'n mens se kanse om borskanker te kry?Noem asseblief soveel risikos\ dinge wat u kan aan dink.*<br><br>**DO NOT READ OUT THE OPTIONS. CIRCLE ALL THAT THE CLIENT MENTIONS DO NOT PROMPT.**		YES	NO	
		a. Family history of breast cancer/ Familie geskiedenis van borskanker	1	0	
		b. Having a past history of breast cancer/Het u 'n geskiedenis in die verlede van borskanker	1	0	
		c. Beginning menopause after age 55/Begin van menopouse na die ouderdom van 55	1	0	
		d. Starting your period before age 11/Begin van maandstonde voor die ouderdom van 11	1	0	
		e. Having children after 30 years of age or not at all/Het kinders na die ouderdom van 30 of glad nie	1	0	
		f. Not breastfeeding/ Nie borsvoeding	1	0	
		g. Using hormonal contraceptive (birth control pills/injection) Gebruik van hormoon voorbehoed (voorbehoedpil, inspuiting)	1	0	
		h. Using hormone replacement therapy/HRT after menopause/ Gebruik van hormonale vervangings terapie na menopouse	1	0	
		i. Drinking alcohol/Alkohol gebruik	1	0	
		j. Being overweight/ Oorgewig	1	0	
		k. Doing little exercise/ Doen van min oefeninge	1	0	
		l. Other (please specify) Ander spesifiseer: ________________________________	1	0	
		m. Don't know/Not sure Weet nie Nie seker	1	0	

No.	Questions and filters	Coding categories	YES	NO	NS	Skip
202.	I am going to read out a list of things. For each can you tell me if this increases a woman's risk of breast cancer?<br>*Ek gaan 'n lys van goed uit lees. Vir elke een wat ek lees kan u vir my sê of dit die risiko van borskanker vermeerder?*<br><br>**GO THROUGH EVERY OPTION AND CIRCLE EITHER 1 IF YES, 0 IF NO OR 3 IF NOT SURE (NS)**		**YES**	**NO**	**NS**	
		a. Family history of breast cancer/ Familie geskiedenis van borskanker	1	0	88	
		b. Having a past history of breast cancer/Het u 'n geskiedenis in die verlede van borskanker	1	0	88	
		c. Beginning menopause after age 55/Begin van menopouse na die ouderdom van 55	1	0	88	
		d. Starting your period before age 11/Begin van maandstonde voor die ouderdom van 11	1	0	88	
		e. Having children after 30 years of age or not at all/Het kinders na die ouderdom van 30 of glad nie	1	0	88	
		f. Not breastfeeding/ Nie borsvoeding	1	0	88	
		g. Using hormonal contraceptive (birth control pills/injection) Gebruik van hormoon voorbehoed (voorbehoedpil, inspuiting)	1	0	88	
		h. Using hormone replacement therapy/HRT after menopause/ Gebruik van hormonale vervangings terapie na menopouse	1	0	88	
		i. Drinking alcohol/Alkohol gebruik	1	0	88	

No.	Questions and filters	Coding categories	Code			Skip
		j. Being overweight/ Oorgewig	1	0	88	
		k. Doing little exercise/ Doen van min oefeninge	1	0	88	
203.	What do you think are signs of breast cancer? Please name as many as you can think of.<br>*Wat dink u is tekens van borskanker? Noem asseblief soveel as moontlik waaraan u kan dink.*<br><br>**DO NOT READ OUT THE OPTIONS. CIRCLE EACH ONE AS THE CLIENT MENTIONS THEM.**<br><br>**WHERE NECESSARY PROMPT CLIENT FOR EXACT ANSWER E.G. IF CLIENT SAYS LUMPS – ASK IF SHE IS REFERRING TO LUMPS IN ANY PARTICULAR PART OF THE BODY**		**Yes**		**No**	
		a. Lumps in the breast / Knoppe in die bors	1		0	
		b. Lumps in the armpit/ Knoppe in die onderarm	1		0	
		c. Bleeding or discharge from the nipple/ Bloeding of afskeiding van die tepel	1		0	
		d. Change in position of the nipple / Verandering in die posisie van die tepel	1		0	
		e. Pulling in of the nipple/ Intrekking van die tepel	1		0	
		f. Rash on/around the nipple/ Uitslag op \of rondom die tepel	1		0	
		g. Wrinkling or dimpling of the breast skin / Rimpeling of induiking van die vel van die bors	1		0	
		h. Redness of the breast skin / Rooihuid van die vel van die bors	1		0	
		i. Change in shape of the breast / Verandering van die struktuur van die voorkoms van die bors	1		0	
		j. Change in size of the breast / Verandering in die grootte van die bors	1		0	
		k. Pain in breast or armpit /Pyn in bors of onderarm	1		0	
		l. Other (please specify)/Ander(spesifieseer asseblief):<br>____________________	1		0	
		m. Don't know/Not sure Weet nie/ Nie seker nie	1		0	
204.	Can you tell me which of these are signs of breast cancer?<br>*Kan u my sê wat van hierdie is tekens van bors kanker?*<br>.<br><br>**GO THROUGH EVERY OPTION AND CIRCLE 1 IF YES, 0 IF NO OR 88 IF NOT SURE (NS)**		**Yes**	**No**	**NS**	
		a. Lumps in the breast / Knoppe in die bors	1	0	88	
		b. Lumps in the armpit/ Knoppe in die onderarm	1	0	88	
		c. Bleeding or discharge from the nipple/ Bloeding of afskeiding van die tepel	1	0	88	
		d. Change in position of the nipple / Verandering in die posisie van die tepel	1	0	88	
		e. Pulling in of the nipple/ Intrekking van die tepel	1	0	88	
		f. Rash on/around the nipple/ Uitslag op \of rondom die tepel	1	0	88	
		g. Wrinkling or dimpling of the breast skin / Rimpeling of induiking van die vel van die bors	1	0	88	
		h. Redness of the breast skin / Rooihuid van die vel van die bors	1	0	88	
		i. Change in shape of the breast / Verandering van die struktuur van die voorkoms van die bors	1	0	88	
		j. Change in size of the breast /Verandering in die grootte van die bors	1	0	88	
		k. Pain in breast or armpit /Pyn in bors of onderarm	1	0	88	
Now I will make statements and ask you whether you strongly agree, agree, are unsure, disagree, or strongly disagree with each statement. / *Nou gaan ek stelings maak en vir u vra of u sterk saamstem,saamstem, onseker, stem nie saam,sterk nie saamstem met elke stelling.*						
205.		**DO YOU/ Wil u:**<br>Strongly agree/ Sterk saam stem	1			

	How much do you agree with this: You only need to get a breast lump checked for cancer if it is painful.<br>*Hoe stem u saam met dit: U het nodig om 'n borsknop te laat ondersoek vir kanker as dit pynlik is.*<br><br>**READ ALL OPTIONS THEN CIRCLE ONE.**	Agree / Saam stem	2	
		Not sure /Onseker	3	
		Disagree / Stem nie saam nie	4	
		Strongly disagree/ Sterk stem nie saam	5	
206.	How much do you agree with this: You only need to get a breast lump checked for cancer if it gets bigger.<br>*Hoe stem u saam met dit: U het nodig om 'n borsknop te ondersoek vir kanker as dit groter raak..*<br><br>**CIRCLE ONE**	**DO YOU/ Wil u:**<br>Strongly agree/ Sterk saam stem	1	
		Agree / Saam stem	2	
		Not sure /Onseker	3	
		Disagree / stem nie saam nie	4	
		Strongly disagree/ sterk stem nie saam	5	
207.	How much do you agree with this: Only women with a family history of breast cancer can get breast cancer.<br>*Stem u saam met dit: Net vroue wat 'n familie geskiedenis van borskanker het kan borskanker kry.*<br><br>**CIRCLE ONE**	**DO YOU/ Wil u:**<br>Strongly agree/ Sterk saam stem	1	
		Agree / stem saam	2	
		Not sure /onseker	3	
		Disagree / Stem nie saam nie	4	
		Strongly disagree/ Sterk stem nie saam nie	5	
208.	How much do you agree with this: Treatment recommended by traditional healers, sangomas, and homeopaths is effective for curing breast cancer.<br>*Stem u saam met dit: Behandeling voorgeskryf deur tradisionele kenners , sangomas en homeopate is effektief om borskanker te genees.*<br><br>**CIRCLE ONE**	**DO YOU/ Wil u:**<br>Strongly agree/ Sterk saam stem	1	
		Agree / Stem saam	2	
		Not sure /Onseker	3	
		Disagree / Stem nie saam nie	4	
		Strongly disagree/ Sterk stem nie saam nie	5	

SECTION 3: Presence of breast cancer risk factors and co-morbidities

READ: "Thank you for sharing that information with me. Now I would like to ask you some questions about your health and family history."/*"Dankie dat u die inligting met my gedeel het. Nou wil ek graag u vrae vra oor u gesondheid en familie geskiedenis.*

No.	Questions and filters	Coding categories	Code		Skip
301.	Does anyone in your family have **breast** cancer now or in the past?<br>*Het enige iemand van u familie borskanker nou of in die verlede?*	Yes/ Ja	1		
		No / Nee	0		→**303**
		Don't know/Not sure weet nie/ nie seker	88		→**303**
302.	Who in your family had/has **breast** cancer?Wie in u familie het borskanker gehad of het borskanker?<br><br>**CIRCLE ALL THAT APPLIES**		Yes	No	
		Mother/Moeder	1	0	
		Sister / Suster	1	0	
		Daughter/ Dogter	1	0	

No.	Questions and filters	Coding categories	Code		Skip
		Aunt, cousin, grandmother, half-sister /Tante/Niggie,ouma, half suster	1	0	
		Other relative(s) / ander familielede. Specify/ Spesifiseer: ________________	1	0	
303.	Have you reached menopause? (periods stopped/change of life)<br>*Het u menopouse bereik? (maandstonde gestop\oorgangsjare)*	Yes/ Ja	1		
		No / Nee	0		→305
304.	Are you currently on hormone replacement therapy (HRT)?<br>*Is u huidiglik op hormonale verwangings terapie?*	Yes/ Ja	1		→306
		No / Nee	0		→306
		Don't know/Not sure Weet nie/ nie seker nie	88		→306
305.	Are you currently pregnant?<br>*Is u nou swanger?*	Yes/ Ja	1		
		No / Nee	0		
306.	Have you given birth to any child(ren)?<br>*Het u geboorte gegee aan enige kind(ers)?*	Yes/ Ja	1		
		No / Nee	0		→309
307.	How many children have you given birth to?<br>*Hoeveel kinders het u aan geboorte gegee?*	☐☐			
308.	Did you ever breastfeed (any of) your child(ren)?<br>*Het u ooit geborsvoed(enige) van u kind(ers)?*	Yes/ Ja	1		
		No / Nee	0		
309.	Have you used any form of **hormonal** contraceptives (family planning—birth control pills, injections, implant, Mirena—not the copper IUD))?<br>*Het u enige vorm van hormonale voorbehoudmiddel - voorbehoed pille, inspuiting, inplanting, Mirena- nie die koper IUA)gebruik?*	Yes/ Ja	1		
		No / Nee	0		
		Don't know/Not sure Weet nie/ Nie seker	88		
310.	Do you currently drink more than a half a glass of wine or a can of beer *or* a tot of spirit a day?<br>*Drink u nou meer as 'n halwe glas wyn of 'n blikkie bier of 'n bietjie spiritis 'n dag ?*	Yes/ Ja	1		→312
		No / Nee	0		
311.	In the past, did you ever drink more than a half a glass of wine or a can of beer or a tot of spirit a day?<br>*In die verlede het u ooit meer as 'n halwe glas wyn of 'n blikkie bier of n bietjie spiritis 'n dag gedrink?*	Yes/ Ja	1		
		No / Nee	0		
312.	Do you currently smoke –cigarettes, hand rolled cigarettes, pipes or cigars?<br>*Rook u nou sigarette, hand gerollde sigarette, pype of sigare?*	Yes/ Ja	1		→314
		No /Nee	0		
313.	Did you ever smoke cigarettes, hand rolled cigarettes, pipes, or cigars in the past?<br>*Het u ooit sigarette gerook, handgerollde sigarette, pype of sigare?*	Yes/ Ja	1		
		No / Nee	0		

			Yes	No	NS	Decl	
314.	Have you ever been told by a doctor, nurse or health care professional that you have […]<br>Was u ooit deur 'n dokter ,verpleegster of profesionele gesondheid werker gesê dat u het [....]<br><br>**GO THROUGH ALL THE OPTIONS AND CIRCLE A RESPONSE FOR EACH.**<br><br>**IF PARTICIPANT DECLINES TO ANSWER, CIRCLE 77.**	a. Benign breast disease or lump/Non-cancerous breast lump/fibroadenoma / Benigne borssiekte of knop/Nie kankeragtige borsknop/fibroadenoom	1	0	88	77	
		b. Tuberculosis (TB)—current infection only/ *Tuberkulose (TB) - huidige infeksie alleenlik*	1	0	88	77	
		c. Hypertension/High Blood Pressure/ Hipertensie\ Hoë bloed	1	0	88	77	
		d. Diabetes/High blood sugar/ Diabetes/ hoë bloed suiker	1	0	88	77	
		e. Stroke/ Beroerte	1	0	88	77	
		f. Heart problems/ Hart probleme	1	0	88	77	
		g. Any other cancer (other than breast)/ Enige ander kanker (nie bors)	1	0	88	77	
		h. Chronic lung disease/ Kroniese long siekte	1	0	88	77	
		i. Chronic kidney failure/ Kroniese nier versaking	1	0	88	77	
		j. Liver disease / Lewer siekte	1	0	88	77	
		k. Arthritis/ Artritis	1	0	88	77	
		l. Ulcers/ Maagsere	1	0	88	77	
		m. HIV/AIDS/ HIV|AIDS	1	0	88	77	

SECTION 4: Breast cancer examinations and pathways to care

READ: "Thank you for sharing that information with me. Now I have a few more questions to understand better your journey to the breast clinic at XXX." / *"Dankie dat u hierdie inligting met my deel. Nou het ek nog 'n paar vrae om u beter te verstaan met u reis na die borskliniek by XXX "*

No.	Questions and filters	Coding categories	Code	Skip
401.	Are you in the habit of checking your breasts for lumps or any other changes?<br>*Is u in 'n gewoonte om u borste te ondersoek vir enige veranderings?*	Yes/ Ja	1	
		No / Nee	0	→**403**
402.	How often would you say you are in the habit of checking your breasts?<br>*Hoe dikwels sal u se het u die gewoonte om u borste te ondersoek?*<br><br>**READ OPTIONS THEN CIRCLE ONE**	At least once a month/ Ten minste een keer per maand	1	
		At least once every 6months/ Ten minste een keer elke ses maande	2	
		At least once a year/ Ten minste een keer per jaar	3	

403.	Did you ever have a clinical breast exam, any time before thebreast changes started? This is when a doctor or nurse feels each of your breasts to look for any lumps or changes.<br>*Het u ooit 'n kliniese bors ondersoek enige tyd gehad voor die bors verandering begin het? Dit is wanneer 'n dokter of 'n verpleegster altwee van u borste ondersoek vir enige knoppe of verandering.*<br><br>**CIRCLE ONE**	Yes/ Ja	1	
		No / Nee	0	→**406**
		Don't know/Not sure Weet niei/ Nie seker	88	→**406**
404.	When was the last time you had a clinical breast exam, before your breast changes started? Would you say it was…<br>Wanneer het u die laaste kliniese bors ondersoek gehad, voor u bors veranderings begin het? Sal u sê dit was...<br><br>**READ ALL OPTIONS AND CIRCLE ONE**	Less than 6 months before my breast changes started/ Minder as 6 maande voor verandering in my bors begin	1	
		Between 6 months and a year before my breast changes started/ Tussen 6 maande en 'n jaar voor verandering in my bors	2	
		More than a year before my breast changes started/ Meer as 'n jaar voor my verandering in die bors begin.	3	
405.	Who did this last clinical breast exam (the exam that happened before current signs were noticed)?<br>*Wie het die laaste kliniese bors ondersoek gedoen (Die ondersoek voor u huidige tekens opgelet is?*<br><br>**READ ALL OPTIONS AND CIRCLE ONE**	Doctor or nurse at a primary health clinic/ Dokter of verpleegster by 'n primêre gesondsheids kliniek	1	
		Nurse at a mobile clinic/ Verpleegster by 'n mobiele kliniek	2	
		Private doctor/ Private dokter	3	
		Doctor or nurse at a public hospital/ Dokter of verpleegster by ;n publieke hospitaal	4	
		Don't know/Not sure/Can't remember/ Weet nie\ Nie seker\ Kan nie onthou nie	88	
406.	Before your visit to the XXX Clinic did you ever have a mammogram?This is when your doctor or health care professional takes an X-ray of your breasts.<br>*Voor u besoek na XXX kliniek het u ooit 'n mammogram gehad?Dit is wanneer 'n dokter of 'n profesionele gesondsheid werker X- trale van u bors neem?*<br><br>**CIRCLE ONE**	Yes/ Ja	1	
		No / Nee	0	→**410**
		Don't know/Not sure Weet nie/ Nie seker nie	88	→**410**
407.	When was the last time you had a mammogram, before your visit to XXX Clinic? Wanneer was die laaste tyd wat u 'n mammogram gehad het, dit is voor u besoek by XXX kliniek?<br><br>**READ OPTIONS AND CIRCLE ONE**	Less than 1 year before my breast changes started. Minder as 1 jaar voordat die verandering in my bors begin het	1	
		Between 1 and 2 years ago before my breast changes started / *tussen 1 en 2 jaar gelede voordat die verandering in my bors begin het*	2	
		More than 2 years before my breast changes started/ Meer as 2 jaar gelede voordat die verandering in my bors begin het	3	

408.	What was your reason for getting that mammogram?<br>*Wat was u rede om daardie mammogram te kry?*<br><br>**DO NOT READ OPTIONS. LISTEN TO THE PARTICIPANT AND CIRCLE ONE.**	Routine check-up - no symptoms / Roetine opvolg- geen tekens	1	
		Routine check-up - I have past disease/ Roetine opvolg - Ek het 'n vorige siekte	2	
		Family history of breast cancer / Familie geskeidenis van borskanker	3	
		Had symptoms (e.g. breast lump) / Het tekens (bv. borsknop)	4	
		Not sure /Nie seker	5	
		Other /Ander. Specify/ Spesifiseer<br>___________________________	6	
409.	Where did you have this mammogram done?<br>*Waar was die mammogram gedoen?*<br><br>**READ OPTIONS AND CIRCLE ONE.**	Public sector hospital/ Publieke hospitaal	1	
		Private doctor/hospital/ Private dokter\ hospitaal	2	
		Other/ Ander. Specify/Spesifiseer:<br>_______________________________________	3	
410.	When did you first notice a change/s in your breast?<br>*Wanneer het u die eerste keer opgelet daar is 'n verandering in u bors?*<br><br>**USE CALENDAR TO GUIDE PARTICIPANT RESPONSE. IF EXACT DAY OF MONTH NOT KNOW, RECORD AS THE 15TH**	___ ___ / ___ ___ / ___ ___<br>DD MM YY\JJ		
411.	What was the **FIRST** change you noticed?<br>*Wat was die* ***EERTSE*** verandering wat u opgelet het?<br><br>**DO NOT READ OPTIONS BUT IF NECESSARY PROMPT FOR SPECIFIC ANSWER e.g. IF PERSON SAYS LUMP/S ASK WHERE IN THE BODY THE LUMP WAS NOTICED.**<br><br>**AFTER PARTICIPANT RESPONDS, CIRCLE ONE**	a. Lump/s in the breast/ Knoppe in bors	1	
		b. Lump/s in the armpit/ Knoppe(in die onderarm)	2	
		c. Bleeding or discharge from the nipple /Bloeding of afskeiding van die tepel	3	
		d. Change in position of the nipple/ Verandering van die posisie van die tepel.	4	
		e. Pulling in of the nipple/ Intrek van tepel	5	
		f. Rash on/around the nipple/ Uitslag op of rondom die tepel	6	
		g. Wrinkling or dimpling of the breast skin/Rimpeling of induiking van die vel van die bors.	7	
		h. Redness of the breast skin/ Rooiheid van vel van bors	8	
		i. Change in shape of the breast/verandering in die vorm van bors	9	
		j. Change in size of the breast/Verandring in groote van bors	10	
		k. Pain in breast or armpit / Pyn in bors of onderarm	11	
		l. Other (please specify)/ Ander (spesifiseer asseblief)<br>_________________________________	12	

412.	What did you think this change meant?<br>*Wat dink u het die verandering beteken?*<br><br>**DO NOT READ OPTIONS.**<br>**CIRCLE ALL THAT APPLIES.**		**Yes**	**No**	
		a. Thought it was not serious/ Gedink dit is nie ernstig	1	0	
		b. Thought it was breast cancer /Gedink dit was borskanker	1	0	
		c. I was not sure / Ek was nie seker	1	0	
		d. Other (please specify) Ander(spesifiseer asseblief)<br>___________________________	1	0	
413.	Apart from (change in 411), did you notice any other changes?<br>*Behalwe van (verandering in 411), het u enige ander veranderings opgelet?*	Yes/ Ja	1		
		No / Nee	0		→**415**
		Don't know/Not sure / Weet nie /nie seker	88		→**415**
414.	What other changes did you notice?<br>*Watter ander veranderings het u opgelet?*<br><br>**DO NOT READ OPTIONS.**<br>**CIRCLE ALL THAT APPLIES**		**Yes**	**No**	
		a. Lump/s in the breast/ Knoppe in bors	1	0	
		b. Lump/s in the armpit/ Knoppe(in die onderarm)	1	0	
		c. Bleeding or discharge from the nipple /Bloeding of afskeiding van die tepel	1	0	
		d. Change in position of the nipple/ Verandering van die posisie van die tepel.	1	0	
		e. Pulling in of the nipple/ Intrek van tepel	1	0	
		f. Rash on/around the nipple/ Uitslag op of rondom die tepel	1	0	
		g. Wrinkling or dimpling of the breast skin/Rimpeling of induiking van die vel van die bors.	1	0	
		h. Redness of the breast skin/ Rooiheid van vel van bors	1	0	
		i. Change in shape of the breast/verandering in die vorm van bors	1	0	
		j. Change in size of the breast/Verandring in groote van bors	1	0	
		k. Pain in breast or armpit / Pyn in bors of onderarm	1	0	
		l. Other (please specify)/ Ander (spesifiseer asseblief)<br>_______________________________	1	0	
415.	After you noticed the changes in your breasts, when did you think you should have it checked?Would you say it was…<br>*Nadat u die veranderings in u bors opgelet het wanneer het u gedink moet u gaan vir 'n ondersoek. Sal u se dit was ...*<br><br>**READ OPTIONS AND CIRCLE ONE**	On the same day you noticed them/ Op dieselfde dag het u dit op gelet	1		
		Within the first 2 weeks of noticing them/ Binne die eerste 2 weke vandat ek dit op gelet het	2		
		Between 2 weeks and a month/ Tussen 2 weeke en 'n maand	3		
		Between 1 and 3 months/ Tussen 1 en 3 maande	4		
		Between 3 and 6 months / Tussen 3 en 6 maande	5		
		More than 6 months / Meer as 6 maande	6		
		Don't know/Not sure / Weet nie / Nie seker	88		

			Yes	No	
416.	What made you think that you should get the symptoms checked?<br>*Wat het u laat dink om die tekens te ondersoek?*<br><br>**ALLOW PARTICIPANT TO RESPOND THEN CIRCLE ALL THAT APPLIES**	a. I felt pain / Ek het pyn gevoel	1	0	
		b. The lump was getting bigger/Die knop het groter geraak	1	0	
		c. My family/friends/relatives told me to have it checked / My familie/ vriende het gesê ek moet dit laat ondersoek	1	0	
		d. I read a pamphlet/attended breast cancer awareness event/watched a TV program/ Ek het n pamflet gelees /het n borskanker bewustheids program bygewoon/ het n tv program gekyk	1	0	
		e. I was worried about the changes/I wanted to be sure nothing was wrong /Ek was bekommerd oor die verandering/ ek wou seker maak niks was verkeerd nie	1	0	
		f. Other (please specify) Ander (spesifiseer asseblief) ________________________________	1	0	
		g. Don't know/Not sure / Ander/Nie seker	1	0	
417.	After deciding you needed to have your breasts checked, when did you first see someone to get your breasts checked? Would you say it was….<br>*Nadat u besluit het dat u , u bors gaan ondersoek wanneer het u vir die eerste keer eimand gesien om u bors te ondersoek. Sal u se dit was...*<br><br>**READ OPTIONS AND CIRCLE ONE**	On the same day you decided you needed your breasts checked / Op dieselfdedag wat u besluit het om u borste te laat ondersoek	1		**→419**
		Within the first 2 weeks of deciding you needed your breasts checked/ Binne die eerste 2 weke toe u besluit het om u borste te ondersoek	2		
		Between 2 weeks and a month / Binne 2 weke en n maand	3		
		Between 1 and 3 months/ Binne 1 en 3 maande	4		
		Between 3 and 6 months / Binne 3 en 6 maande	5		
		More than 6 months / Meer as 6 maande	6		
		Don't know/Not sure / Weet nie /Nie seker	88		
418.	What was the reason for waiting for (***interviewer state time mentioned in previous questions)***between deciding to seek help, and your actual appointment date.<br>*Wat was die rede vir die wag (onderhoudvoerder skryf tyd neer wat in vorige vraag genoem is) intussen om te besluit om hulp te soek en u afspraak datum.*<br><br>**ALLOW CLIENT TO RESPOND THEN CIRCLE ALL THAT APPLIES.**<br><br>**DO NOT PROMPT.**		**Yes**	**No**	
		a. That was the earliest appointment available/ Dit was die vroegste afspraak beskikbaar	1	0	
		b. I was busy with work and could only attend then / Ek was besig om te werk en kon eers toe bywoon	1	0	
		c. I had to take care of my family/other family responsibilities / Ek moes na my familie omsien/ander familie verantwoordelikheide	1	0	
		d. I did not have money for transport / Ek het nie geld gehad vir vervoer nie	1	0	
		e. The clinic was too far / Die kliniek was te vêr	1	0	
		f. I did not know where to go/ Ek het nie geweet waar om te gaan nie	1	0	
		g. I thought it would get better / Ek het gedink dit sou beter word	1	0	
		h. I was scared/ Ek was bang	1	0	
		i. Other (please specify) Ander (spesifiseer asseblief) ________________________________	1	0	

419.	Where did you FIRST go to have your breast checked?<br>*Waar het u EERSTE gegaan om u bors te ondersoek?*<br><br>**CIRCLE ONE. IF UNSURE WHAT TYPE, NOTE THE NAME OF THE FACILITY AND CONSULT PI FOR CODING.**	Health care provider at clinic or day hospital/ Gesondheid werker by die kliniek of daghospitaal	1	
		Health care provider at a secondary/district hospital (not day hospital)/ Gesondheid werker by n sekondêre/distrik hospitaal (nie daghospitaal)	2	
		Private GP or specialist/ Private dokter of spesialis	3	
		Homeopath/ Homeopaat	4	
		Traditional healer/ Tradisionele kenner	5	
		Other (please specify) Ander (spesifiseer asseblief)___________________________________	6	
420.	Please think back to all the visits you made to any type of health provider including traditional and herbal healers. How many visits in total did you make before arriving at the XXX Breast Clinic?<br>*Dink terug na all u besoeke wat u gehad het na enige tipe gesondheidsorg werker insluitende tradisionele kenner en kruiie kenner. Hoeveel besoeke in totaal het u gemaak voordat u by XXX klinik aangekom het?*<br><br>**CHECK PARTICIPANT IS COUNTING ALL: TRADITIONAL HEALER+ GP + CLINIC+ ANY OTHER PROVIDER**	☐☐ visits/ besoeke		

<table>
<tr>
<td rowspan="2">421.</td>
<td rowspan="2">Can you tell me your journey from the first person you saw to have our breast checked until the time you came to breast clinic? I am interested in who you saw, when and what happened at each stop along the way.
Kan u my vertel van u reis vanaf die eerste persoon wat u gesien het om u bors te ondersoek tot die tyd dat u na die borskliniek gekom het? Ek is geintereseerd in wie u gesien het, wanneer en wat het gebeur met elke besoek....

COMPLETE THE TABLE IN THE ORDER OF VISITS FROM FIRST TO LAST VISIT (<u>XXX Breast Clinic</u>), ADDING MORE ROWS IF NECESSARY AND RELEVANT ADDITIONAL NOTES.

IF EXACT DATE UKNOWN, ENTER 15 FOR DAY.</td>
<td>INTERVIEWER</td>
</tr>
<tr>
<td>
<table>
<tr><th>Type of provider seen</th><th>Type of facility</th><th>Date</th><th>What happened at visit</th></tr>
<tr><td>1.</td><td></td><td></td><td></td></tr>
<tr><td>2</td><td></td><td></td><td></td></tr>
<tr><td>3</td><td></td><td></td><td></td></tr>
<tr><td>4</td><td></td><td></td><td></td></tr>
<tr><td>5</td><td></td><td></td><td></td></tr>
<tr><td>6</td><td></td><td></td><td></td></tr>
</table>
Additional notes:
</td>
</tr>
</table>

READ: "We have come to the end of this interview. Thank you for your time. The information you have shared has been very helpful."/"Ons het aan die einde van die onderhoud gekom dankie vir u tyd. Die inligting wat u met my gedeel het is van groot hulp"

DO NOT READ TO PARTICIPANT:

<table>
<tr><td rowspan="2">422.</td><td rowspan="2">Did the participant have an accompanying person assisting in their interview (i.e. a friend or family member)?</td><td>Yes</td><td>1</td><td></td></tr>
<tr><td>No</td><td>0</td><td></td></tr>
</table>

SECTION 5: To be completed from clinical records

No.	Questions and filters	Coding categories	Code	Skip
501.	Date Section 5 completed	___ ___ / ___ ___ / ___ ___<br>DD MM YY		
502.	Income status	H0	0	
		H1	1	
		H2	2	

No.	Questions and filters	Coding categories			Code	Skip
		H3			3	
		Not recorded (NR)			99	
503.	Date first seen at breast clinic (*typically a Friday)*	___ ___/ ___ ___/ ___ ___ DD MM YY				
504.	Date seen at combined breast clinic (*typically a Wednesday)*	___ ___/ ___ ___/ ___ ___ DD MM YY				
505.	Clinical examination:		Yes	No	NR	
		a. Lump	1	0	99	
		b. Oedema	1	0	99	
		c. Nipple retraction	1	0	99	
		d. Ulceration	1	0	99	
		e. Skin dimpling	1	0	99	
506.	If lump present (505a=1): Size	☐☐.☐ cm **X** ☐☐.☐ cm			*If only one measurement, record in first set of boxes*	
507.	Initial cytology date	___ ___/ ___ ___/ ___ ___ DD MM YY				
508.	Initial Cytology: cytology number (SCY)	☐☐☐☐☐☐☐				
509.	Initial Cytology result	C1 Inadequate/Not representative			1	
		C2 Benign			2	
		C3 Atypia favour benign			3	
		C4 Atypia Favour Malignant			4	
		C5 Malignant(e.g. positive for carcinoma)			5	
510.	Histology/biopsy date	___ ___/ ___ ___/ ___ ___ DDMMYY				
511.	Histology number: SCA	☐☐☐☐☐☐☐				

512.	Histology/Biopsy Result	B1 Inadequate/Not representative	1	
		B2 Benign	2	
		B3 Atypia favour benign	3	
		B4 Atypia Favour Malignant	4	
		B5 Malignant	5	
513.	Histological subtype	DCIS (Ductal carcinoma in situ): **low grade**	1	
		DCIS (Ductal carcinoma in situ): **intermediate grade**	2	
		DCIS (Ductal carcinoma in situ): **high grade**	3	
		LCIS (lobular carcinoma in situ)	4	
		Invasive: **ductal**	5	
		Invasive: **lobular**	6	
		Invasive: **other**	7	
		Primary Lymphoma	8	
		Angiosarcoma	9	
		Malignant Phylloides	10	
		Carcinosarcoma/ metaplastic	11	
514.	Receptor status: ER	☐ out of 8<br>*If not recorded, record as "9."*		
515.	Receptor status: PR<br>*If ER negative*	Positive	1	
		Negative	2	
		Not recorded	99	
516.	Receptor status: HER2	1+	1	
		2+	2	
		3+	3	
		Not recorded	99	
517.	Mammogram date	___ ___ / ___ ___ / ___ ___<br>DDMMYY		
518.	Mammogram number (accession number)	☐☐☐☐☐☐☐☐		
519.	Mammogram results: birads score<br>*(if two results—one for each breast—are recorded, please code for the most severe, i.e. the highest birads score)*	0 Additional imaging needed	0	
		1 Normal	1	
		2 Benign	2	
		3 Most likely benign but follow up imaging or biopsy needed	3	
		4 Suspicious findings	4	
		5 Highly suspicious for malignancy	5	
		6 Confirmed malignancy on biopsy	6	

520.	Consensus clinical stage at diagnosis – TNM<br>a. T	T1mic (tumour in situ);	0	
		T1 (includes T1a; ; T1b; T1c)	1	
		T2;	2	
		T3;	3	
		T4(includes T4a; T4b; T4c; T4d)	4	
	b. N	N0	0	
		N1	1	
		N2	2	
		N3	3	
		Nx	4	
	c. M	M0	0	
		M1	1	
		MX	2	
521.	HIV status	HIV positive	1	
		HIV negative	2	
		Not recorded	99	
	**Management plan: First treatment planned**			
522.	Surgery	Yes	1	
		No	0	→523
	a. Breast surgery type	Breast surgery: wide local excision (**WLE)**	1	
		Breast surgery: mastectomy (no reconstruction)	2	
		Breast surgery: mastectomy and reconstruction	3	
	b. Axilla surgery type	Axilla surgery: sentinel lymph node biopsy (**SLNB**)	1	→526
		Axilla surgery: axillary node clearance (**ANC)**	2	→526
		Axilla surgery: none	3	→526
523.	Chemotherapy	Yes	1	
		No	0	→524
	a. Chemotherapy type	Chemotherapy: Neoadjuvant	1	→526
		Chemotherapy: Palliative	2	→526
524.	Hormonal treatment	Yes	1	
		No	0	→525
	a. Hormonal treatment type	Hormonal treatment: Neoadjuvant	1	→526
		Hormonal treatment: Palliative	2	→526
525.	Radiotherapy	Yes	1	
		No	0	
	a. Radiotherapy type	Radiotherapy: Neoadjuvant	1	
		Radiotherapy: Palliative	2	

526.	Date first treatment (will commence)	___ ___ / ___ ___ / ___ ___<br>DD MM YY		
