# Supplementary material for: From symptom discovery to treatment - women's pathways to breast cancer care: a cross-sectional study
Source: BMC Cancer. 2018 Mar 21;18:312. doi: 10.1186/s12885-018-4219-7 (PMC5863383; doi:10.1186/s12885-018-4219-7)
Supplement: Supplementary file 2 — Predictors of the Patient Interval. Table with results of the Cox Regression analysis (DOCX 17 kb) [file 12885_2018_4219_MOESM2_ESM.docx]

**Additional file 2: Predictors of the Patient Interval (n = 166)**

| **Variable** | **Hazard ratio (95% CI)** | **P-value** |
| --- | --- | --- |
| Age > 54 years | 0.59 (0.40 - 0.86) | 0.007 |
| Education level (ref. < Grade 8) | | |
| Grade 8 - 11 | 1.18 (0.75 - 1.86) | 0.469 |
| Grade 12 + | 1.04 (0.63 - 1.72) | 0.885 |
| Marital status (ref. married) | | |
| Single in stable relationship | 1.26 (0.45 - 3.51) | 0.657 |
| Single | 1.55 (0.97 - 2.46) | 0.065 |
| Widowed | 1.25 (0.76 - 2.04) | 0.382 |
| Divorced/separated | 1.26 (0.76 - 2.10) | 0.371 |
| Unprompted composite knowledge (ref. = no knowledge) | | |
| Very little | 0.76 (0.38 - 1.49) | 0.421 |
| Little | 1.21 (0.48 - 3.06) | 0.684 |
| History of co-morbidities (ref. no co-morbidity) | | |
| Benign breast disease | 1.63 (0.90 - 2.93) | 0.105 |
| Any other co-morbidity | 1.25 (0.86 - 1.81) | 0.247 |
| First change breast lump (ref. other) | 1.09 (0.73 - 1.63) | 0.681 |
| Appraisal of first change | | |
| Breast cancer | 1.38 (0.88 - 2.18) | 0.161 |
| Not serious/minor | 1.14 (0.77 - 1.67) | 0.522 |
| Denial | 0.43 (0.19 - 0.97) | 0.042 |
| Reason for seeking care | | |
| Lump getting bigger | 0.51 (0.33 - 0.77) | 0.002 |
| Prompted by family/friends | 1.09 (0.74 -1.60) | 0.673 |
| Wanted to make sure nothing was wrong | 1.76 (1.20 - 2.58) | 0.004 |
| Late stage disease (3 &4) at presentation | 0.85 (0.57 - 1.27) | 0.437 |

CI = Confidence Interval

Ref. = referent
