# Supplementary material for: From symptom discovery to treatment - women's pathways to breast cancer care: a cross-sectional study
Source: BMC Cancer. 2018 Mar 21;18:312. doi: 10.1186/s12885-018-4219-7 (PMC5863383; doi:10.1186/s12885-018-4219-7)
Supplement: Supplementary file 3 — Predictors of the Diagnostic Interval. Table with results of the Cox Regression analysis (DOCX 15 kb) [file 12885_2018_4219_MOESM3_ESM.docx]

**Additional file 3: Predictors of the Diagnostic Interval (n= 182)**

| **Variable** | **Hazard ratio (95% CI)** | **P-value** |
| --- | --- | --- |
| Age > 54 years | 1.04 (0.72 - 1.49) | 0.838 |
| Education level (ref. < Grade 8) | | |
| Grade 8 - 11 | 0.91 (0.60 - 1.38) | 0.661 |
| Grade 12 + | 1.00 (0.63 - 1.60) | 0.993 |
| History of co-morbidities (ref. no co-morbidity) | | |
| Benign breast disease | 0.64 (0.39 - 1.07) | 0.090 |
| Any other co-morbidity | 0.67 (0.47 - 0.96) | 0.029 |
| First change breast lump (ref. other) | 1.27 (0.90 - 1.78) | 0.173 |
| Appraisal of first change | | |
| Not serious/minor | 0.81 (0.59 - 1.11) | 0.199 |
| Denial | 4.61 (1.80 - 11.77) | 0.001 |
| Private sector provider seen first (ref. public) | 0.92 (0.64 - 1.33) | 0.668 |
| Late stage disease (3 &4) at presentation | 1.04 (0.71 - 1.54) | 0.837 |

CI = Confidence Interval

Ref. = referent
