# Supplementary material for: From symptom discovery to treatment - women's pathways to breast cancer care: a cross-sectional study
Source: BMC Cancer. 2018 Mar 21;18:312. doi: 10.1186/s12885-018-4219-7 (PMC5863383; doi:10.1186/s12885-018-4219-7)
Supplement: Supplementary file 4 — Predictors of the Pre-Treatment Interval. Table with results of the Cox Regression analysis (DOCX 14 kb) [file 12885_2018_4219_MOESM4_ESM.docx]

**Additional file 4: Predictors of Pre-Treatment Interval (n = 181)**

| **Variable** | **Hazard ratio (95% CI)** | **P-value** |
| --- | --- | --- |
| Age > 54 years | 1.06 (0.77 - 1.46) | 0.723 |
| Education level (ref. < Grade 8) | | |
| Grade 8 - 11 | 0.68 (0.45 - 1.01) | 0.058 |
| Grade 12 + | 0.88 (0.56 – 1.38) | 0.572 |
| First treatment surgery (ref. other) | 1.47 (0.93 – 2.33) | 0.100 |
| Late stage disease (3 &4) at presentation | 1.78 (1.15 - 2.76) | 0.010 |

CI = Confidence Interval

Ref. = referent
